# Leveraging an implementation science partnership network to understand how Federally Qualified Health Centers operationalize and address health equity

**DOI:** 10.1093/tbm/ibad046

**Published:** 2023-08-05

**Authors:** Kelly A Aschbrenner, Jennifer L Cruz, Gina R Kruse, Huy Nguyen, Cristina Huebner Torres, Maria Celli, Carrie Sarcione, Deepinder Singh, Karen M Emmons

**Affiliations:** Geisel School of Medicine at Dartmouth College, Hanover, USA; Dartmouth Health System, Lebanon, USA; Harvard T.H. Chan School of Public Health, Boston, USA; Massachusetts General Hospital, USA; DotHouse Health, Boston, MA, USA; Caring Health Center, Springfield, MA, USA; Brockton Neighborhood Health Center, Brockton, MA, USA; Dartmouth Health System, Lebanon, USA; Massachusetts General Hospital, USA; Harvard T.H. Chan School of Public Health, Boston, USA

**Keywords:** health equity, Community Health Centers, implementation science, practice partnerships, evidence-based interventions, qualitative methods, learning communities

## Abstract

Health equity-focused implementation research requires using definitions and approaches that are relevant and meaningful to implementation partners. We examined how health equity was operationalized and addressed at Federally Qualified Health Centers (FQHCs). We conducted semi-structured interviews with leadership (*n* = 19) and staff (*n* = 12) at 10 FQHCs in an implementation science partnership network for cancer control equity to understand how they operationalized and addressed health equity. We performed rapid qualitative analysis and shared findings with a larger group of 13 community health centers (including the 10 FQHCs) at an Implementation Learning Community (ILC) to identify action areas for research and practice, followed by a second phase of synthesizing qualitative codes into themes and mapping themes onto a framework for advancing health equity in healthcare organizations. Participants defined health equity as central to the mission of FQHCs, and identified barriers (e.g. financing models) and facilitators (e.g. interpreter services) to advancing health equity at FQHCs. These findings resonated with ILC participants who emphasized the challenge of addressing root cause social determinants of inequities using limited available resources in FQHCs and the importance of developing meaningful collaboration with communities for data collection, data interpretation, data use, and data ownership. Themes captured recommendations to advance health equity in daily work at FQHCs, including investments in staffing, training, and resources. Mapping qualitative themes from health equity-centered interviews with FQHC partners onto a framework for advancing health equity in healthcare organizations can provide clear, context-specific direction for actions aimed at improving health and healthcare equity.

## Teaser

Federally Qualified Health Centers (FQHCs) shared their definitions of health equity, articulated how it was addressed in their practice settings, and identified challenges and opportunities for health equity-focused implementation research and practice.

**Implications**

**Practice:** Federally Qualified Health Centers (FQHCs) are essential partners during project conceptualization and evaluation for health equity-focused implementation research in these settings.

**Policy:** FQHC leaders and staff emphasized the need to sharing de-identified data with community leaders who can shape policy to advance health equity.

**Research:** Conducting formative research to understand how implementation partners operationalize and address health equity can lay a foundation for future health equity-focused implementation research.

## Introduction

Federally Qualified Health Centers (FQHCs) are safety net organizations that fill a critical need for uninsured, underinsured, and other medically underserved patients in the USA by providing services regardless of an individual’s ability to pay. In 2021, FQHCs provided comprehensive primary care, including cancer prevention and screening services, to more than 30 million people ^[1]^. However, FQHCs experience many challenges delivering cancer preventive care services, including inadequate reimbursement, workforce attrition ^[2]^, and patient self-reported barriers such as limited knowledge about cancer screening, fear or worry about the procedure, financial difficulties related to insurance coverage and the cost of testing, and logistical challenges such as transportation and time pressures ^[3, 4]^. These multi-level barriers to early identification and treatment contribute to cancer health disparities among racial and ethnic minoritized populations ^[5]^ and other medically underserved groups ^[6]^ who are more likely to receive care at FQHCs ^[7]^.

As FQHCs seek to close gaps in cancer prevention care, there is a need to understand how to reach patients more equitably with evidenced-based interventions (EBIs) in cancer prevention and control. Health equity is the principle underlying a commitment to reduce and ultimately, eliminate a health disparity and its determinants, including social determinants ^[8–10]^. As a field prioritizing a focus on health equity ^[11]^, implementation science is well positioned for partnered approaches to develop and test strategies for closing gaps in delivery, access, use, and benefit from EBIs, including cancer prevention and control interventions. There have been numerous calls for implementation science investigators to focus on health equity in implementation research ^[11–13]^. These calls have been, in part, driven by concerns that broader efforts to implement EBIs may be further driving inequities ^[14]^, with limited reach and benefit to groups that systematically experience social and structural barriers to healthcare ^[15]^.

Opportunities for health equity-focused implementation science includes partner-engaged cancer control and prevention research that collaboratively identifies and leverages practice partners’ knowledge of inequities and patient-centered solutions used to address them in real world practice ^[16]^. Using definitions, measures, and approaches to promoting health equity that align with our partners’ knowledge and expertise, priorities, policies, and needs and assets can help advance equitable implementation of EBIs ^[17]^. Despite the instrumental role of FQHC’s in advancing health equity, little attention has been given to how professionals employed at FQHCs operationalize strategies to improve health equity. In general, this type of formative research on how implementation partners define and address health equity is not done consistently in implementation science and thus, there is high potential for researchers to misalign strategies at cross-purposes with partners. Understanding how FQHC leadership and staff advance health equity in their daily work is a critical foundation for future partnered research where investigators and FQHCs work together to increase the relevance and impact of health equity-focused implementation research in community settings ^[18]^.

### Purpose of the present study

The purpose of the present study was to: (i) identify how multi-level practice partners at FQHCs (e.g. leaders, providers, and quality improvement and population health staff) defined, measured, and addressed health equity, both broadly and with respect to cancer prevention and screening efforts; (ii) use a facilitated process at an Implementation Learning Community (ILC) to: (a) share qualitative findings from interviews with FQHC leadership and staff with a broader group of FQHCs and explore how the findings resonated with this group and (b) collaboratively identify gaps, challenges and opportunities to advance health equity in FQHCs using implementation science; and (iii) identify themes from the synthesis of qualitative codes and illustrate how these themes map onto an existing framework for advancing health equity in healthcare organizations.

## Methods

### Overview

We conducted this study at the Harvard Implementation Science Center for Cancer Control Equity (ISCCCE), which is funded by the National Cancer Institute as one of seven Implementation Science Centers in Cancer Control (ISC^3^) nationwide ^[19]^. Funded in 2019–2020 by the Cancer Moonshot, an investment in funding by the U.S. government focusing on areas of cancer research with high potential for patient impact, ISC^3^ supports and advances the rapid development, testing, and refinement of innovative approaches to implement a range of EBIs across the cancer control continuum ^[19]^. Improving health equity among underserved populations through implementation science was a theme area highlighted in the ISC^3^ Funding Opportunity Announcement (RFA-CA-19-006) and health equity is an explicit focus of implementation research studies conducted at ISCCCE. The Harvard Longwood Campus IRB, the Dartmouth Health Human Research Protection Program (IRB) and the Mass General Brigham IRB independently reviewed study procedures and determined the research to be minimal risk to human subjects and exempt from ongoing IRB oversight. We followed the Consolidated Criteria for Reporting Qualitative Studies (COREQ) reporting guidelines in preparing this manuscript ^[20]^.

### Setting

ISCCCE has an established community partnership with the Massachusetts League of Community Health Centers (MLCHC), a Primary Care Association that provides support and technical assistance to a network of FQHCs across Massachusetts. Guided by community-engaged research principles ^[21]^, our implementation science partnership aims to engage FQHCs across the state in implementing evidence-based cancer prevention and control interventions to address long-standing inequities in cancer outcomes. An implementation laboratory (I-Lab) facilitates partnerships between a multi-disciplinary team of ISCCCE researchers and MLCHC leadership and FQHC partners to accelerate the integration of implementation science research and capacity building into FQHCs ^[22]^.

The I-Lab hosts a quarterly ILC that provides opportunities for engagement and relationship development among researchers and FQHC partners. The ILC program includes online learning tools and 2-h virtual sessions that include brief presentations by FQHCs participating in implementation pilots describing implementation successes, challenges, and lessons learned; expert speakers on implementation-related topics; moderated panels of implementation teams and investigators; and breakout group discussions facilitated by ISCCCE team members and FQHC partners to integrate the speaker or panel topics with FQHC implementation priorities. All staff of participating FQHCs are invited to attend ILC sessions, including chief executive officers and chief medical officers, quality improvement staff, population health managers, clinicians, medical assistants, peer navigators, and community health workers.

### Participant recruitment and procedures

Participant recruitment for this cross-sectional qualitative study began in July 2021 and the interviews were completed in January of 2022. For the purposes of this study, FQHC employees who worked primarily in a staff-and-operations oversight role within a specific department of the FQHC or FQHC as a whole were classified as leadership, while all other FQHC employees interviewed were classified as staff. Our research team worked directly with MLCHC leadership to generate a list of key informants at FQHCs to invite to participate in one-time qualitative semi-structured interviews. MLCHC leadership sent an email with a brief description of the study to FQHC leadership on the outreach list. Research staff then followed-up with key informants by email to invite them to participate in the interview and scheduled a time for the interview to take place. The research team used a snowball sampling approach ^[23]^ to recruit FQHC staff referred by leadership for interviews. In total, we invited 46 key informants to participate in the interviews. Participants were compensated with a $50 gift card for participation in the 30–45 min interviews. The interviews were conducted by the lead author (KA) and three members of the research team (JC, CS, and DS separately over Zoom). The interviews were audio-recorded, and the study coordinator sent the audio-recordings to a professional transcription service that generated de-identified transcripts for the research team.

### Design

#### Qualitative interview guide

We used a semi-structured interview guide to collect data from individual participants during the one-on-one interviews. The Framework for Advancing Health Equity ^[18]^ provided a conceptual basis for the interview guide. This framework proposes that advancing health equity in diverse healthcare settings requires intentionality; each worker at every level needs to know how to operationalize interventions to advance health equity in their daily jobs. We included questions in the interview guide asking FQHC leadership and staff to describe how they defined, measured, and addressed health equity in their routine practice with an emphasis on health equity in cancer prevention and control. Consistent with the “Creating a Culture of Equity” component of the Framework, we asked FQHC participants to share the extent to which health equity was a priority at their organization in general and specifically in cancer prevention and control. The guide also included questions about participants’ perspectives and experiences with challenges to measuring and addressing inequities in care as well as opportunities for policy and practice innovations to advance health equity in FQHCs.

#### Phase 1—qualitative analysis

We conducted a two-phase framework analysis to facilitate rapid return of results to stakeholders ^[24, 25]^. This approach involved preliminary coding over a 3-month period (March to May 2022) followed by further classification and synthesis of major topics, ideas, and patterns in the data into themes (September to December 2022) ^[26]^. Interview transcripts were managed and coded in NVivo ^[27]^. During first cycle coding in preparation for the ILC, two coders (DS and CS) conducted descriptive, deductive coding to summarize the data from FQHC interviews using a pre-determined codebook developed by the lead author (KA), and second author (JC) based on research questions and domains from the interview guide. The researchers used an iterative process to independently code transcripts ^[26]^. Throughout the coding process, the researchers met after coding batches of five transcripts to review codes, discuss and resolve any disagreements about codes and code meaning, and agree on a final set of codes ^[28]^.

#### Implementation Learning Community

We presented the results of the first phase of the qualitative data analysis to FQHC leadership and staff, including both interview participants and non-participants, at an ILC session held in June of 2022 for the purposes of: (i) sharing and exploring how the findings resonated with this group, and (ii) identifying gaps, challenges and opportunities to advance health equity in FQHCs with implementation research and practice. The lead author (KA) presented the descriptive codes and code categories from the qualitative data analysis. Following the presentation, three FQHC leaders facilitated separate breakout groups using a structured facilitator’s guide that asked participants to: (i) discuss the study results focusing on their reactions to the findings; (ii) identify actionable areas in the results for implementation research and practice; and (iii) to share their thoughts on what would be needed to advance health equity in these areas at FQHCs. Volunteers from each of the three breakout groups reported the main discussion points and ideas generated from their respective breakout groups to the larger group. The large group discussion was used to reach consensus on key considerations for health equity-focused implementation research and practice at FQHCs. Research staff took detailed notes during the breakout groups and the large group discussion, focusing on: (i) participants' overall reactions to study findings; (ii) actionable areas identified in the results; and (iii) what participants' recommended to advance health equity in these areas. The notes did not contain any identifying information and were not formally analyzed. The ILC Zoom session was audio-recorded and available for the research staff to reference if they needed to enhance the session notes.

#### Phase 2—qualitative analysis

In the second phase of qualitative data analysis, the research team conducted an in-depth thematic analysis by grouping categories from the Phase 1 qualitative data analysis into themes. The lead author (KA) initiated this process by condensing the categories from the Phase 1 qualitative data analysis into salient themes that other members of the research team, which included FQHC partners, reviewed and refined with feedback. The identification of themes was also informed by key considerations for health equity-focused implementation research and practice identified during the ILC. Once the themes were identified, the lead author (KA) mapped the themes onto six components of the Framework for Advancing Health Equity ^[18]^: (i) intentionally advance health equity; (ii) create a culture of equity; (iii) operationalize advancing health equity in daily work; (iv) implement roadmap to reduce disparities; (v) payment reform; and (vi) cross-sector partnerships. The research team reviewed and refined the results of the mapping ^[26]^. All co-authors discussed and approved the final set of themes applied to the Framework for Advancing Health Equity.

## Results

### Participants

Our team invited 46 FQHC leadership and staff to participate in qualitative interviews. Among those invited, 31 FQHC employees agreed to participate in the interviews including 19 leadership and 12 staff. The professional roles of FQHC leadership and staff participants are presented in Table 1.

**Table 1:** FQHC leadership and staff participants in semi-structured interviews

Leadership (*n* = 19)	Staff (*n* = 12)
Chief Executive Officer (*n* = 1)	Family Provider (*n* = 1)
Chief Operating Officer (*n* = 1)	Nurse Practitioner (*n* = 1)
Chief Medical Officer (*n* = 4)	Clinical Assistant (*n* = 1)
Chief Clinical Officer (*n* = 1)	Community Health Worker (*n* = 1)
Director of Nursing (*n* = 1)	Community Resource Specialist (*n* = 1)
Chief Quality Officer (*n* = 2)	Behavioral Health Provider (*n* = 1)
Medical Director (*n* = 2)	Prevention Services Manager (*n* = 1)
Chief of Research and Health Officer (*n* = 1)	Practice Transformation Manager (*n* = 1)
QI Director (*n* = 3)	Pop Health Manager (*n* = 1)
Population Health Director (*n* = 1)	QI Manager (*n* = 1)
Director of Community Outreach (*n* = 1)	Grants Manager (*n* = 1)
Pharmacy Director (*n* = 1)	Business Process Analyst (*n* = 1)

#### Phase 1—qualitative categories

FQHC participants defined health equity as central to the mission of FQHCs as safety net organizations delivering care to medically underserved populations. Participants identified barriers (e.g. financing models) and facilitators (e.g. interpreter services) to advancing health equity at FQHCs. Qualitative code categories related to the following interview topics: how health equity is defined at FQHCs; how health equity is measured at FQHCs; how health equity is addressed at FQHCs; challenges to measuring and addressing health equity at FQHCs; and resources, policy, and practice innovations to advance health equity at FQHCs. The descriptive codes within each of these categories along with illustrative quotes by participant type are presented in Table 2.

**Table 2:** Interview topic areas and related codes and illustrative quotes by participant type

Interview topic area	Codes	Sample text
How health equity is defined at FQHCs	Focusing on underserved populations	“*Community health centers have always been focused on health equity. And that’s why we exist—in our mission statement it talks about removing barriers to healthcare, which could be financial, language, cultural. So I think for decades, health centers haven’t really used the word health equity, but that’s why we exist*.” Leader
	Equitable access to healthcare	“*Health equity would be where everyone, all our patients and community members, had the same opportunities and resources. I don’t want to say the same. Had the correct or most appropriate-to-them level of access, resources and opportunities to be as healthy as they would like to be.*” Staff
	Providing person-centered healthcare	“*We define it as offering patients what they need. And we spend a lot of time educating our staff on the difference between equal care and equitable care, that equal care doesn’t necessarily meet everybody’s needs. So kind of that customization, fine-tuning to maximize health*.” Leader
	Identifying and understanding root causes of inequities	“*Health equity in the way that we address it in my work is looking at the root causes of why there are such differences in health outcomes and health concerns among populations. Whatever separating demographics you’re looking at, there are always differences. And often that has to do with things that are more systemic, environmental, things like that are commonly not viewed as health or health indicators…*” Staff
	Opportunities for health and wellness	“*It’s primarily about having opportunities for health and wellness for the community. For instance, in the community, and really meeting our patients where they’re at and meeting their needs. We had this traditional thought before that it was primarily about access, and if you make it available, that was good enough. And we’ve learned over the years that’s really not good enough and that access doesn’t equal equity.*” Leader
How health equity is measured at FQHCs	Electronic medical/health record	“*The electronic medical record provides a lot of data. When we’re looking at our workflows and trying to understand where things break down and how to improve on processes, we look at who’s doing what, and we can measure it by that. And then we can look at it by provider level, and further down by team level, so by medical assistants or by nurses as well.*” Leader
	Quality measures	“*In our patient population it’s really impossible to make progress in any [quality] measures…like hypertension control, child immunizations, colorectal cancer screening rates, cervical cancer screening rates ... if we’re not looking at, ‘Okay, who’s falling in the gap, and why could that be? And how do we need to adjust our approach to meet those patients?’ So in some areas, we’re familiar that there’s larger racial and ethnic inequities than others*.” Leader
	Patient surveys and questionnaires	“*Another thing that we do is qualitative and quantitative patient surveys to see if anybody’s experience is different. Maybe they’re getting the same breast cancer screening, but what is their experience of care?*” Leader
	Social determinants of health screening	“*When we first rolled out our health-related social needs screening we also changed our process for depression screening, developmental screening. A lot of what I was looking at was the screening rates among different populations. We tend to focus a lot on language*.” Staff
	Clinical observations	“*A lot of it comes from what providers and staff observe. There are a lot of gaps that providers and staff will bring up that we address through our case management and nurses*.” Leader
How health equity is addressed at FQHCs	Set improvement targets	“*FQHCs are not always positioned to operate at the level that we aim to operate because we’re impacted by the same structural inequities as our patients. So we set targeted incremental goals to work towards those benchmarks, making center improvements to try and improve those things along the way. And then, we also tailor our goals and our benchmarks based on the lived experience of our provider teams and feedback from our patients and community in order to accommodate and account for the things that are important to them or that they observe*.” Leader
	Tailor and/or adapt patient outreach and engagement strategies	“*We’ll share [data] with our clinical groups, and then it’s about what other strategies for us to outreach or engage patients. So it’s looking at best practices, learning from other sites how they do things, or just tweaking a little bit of what we do already. So doing different test [PDSA] cycles, meaning it’s almost like oversampling; we just over-outreach in the different groups that are a little behind, and then from there you gather some sort of qualitative information, like why are people declining and then tweak and/or tailor further*.” Leader
	Interpreter services	“*We’re looking at how to centralize [language] services here, so we don’t just have to use the translator on the stick because a lot of people are more comfortable with somebody in person that they can look at and say,* “*‘This is what I’m experiencing,*”*’ in their own language, and have somebody be able to translate right in front of them as opposed to through a device where they don’t get to see the person*.” Staff
	Community health workers	“*We have team advocates and community health workers, both do a 360 evaluation of the patient and ensure they’re referring them to the organizations that can provide support.*” Staff
	Transportation	“*Through direct relief, we’ve got 500 Uber rides for people who otherwise can’t get to the vaccine clinics, things like that.*” Staff
	Community resources	“*We have an affiliated gym and grocery store. We have an agreement with an early education center*.” Staff
Challenges to measuring and addressing health equity at FQHCs	Financing model	“*You don’t get paid for addressing health equity. It’s not a billable service. So preventing something from happening, you don’t necessarily get paid for doing that*.” Staff
	Data collection	“*We realize that there’s either inaccurate labeling of patients or a lack of understanding from frontline staffas to what various things mean and why they’re important. One of the limits to our ability to parse out our data in an equity sense is that we don’t necessarily categorize people in the way that they would categorize themselves.*” Leader
		*"We don’t have a great space other than the patient rooms for privacy. So when staff aren’t comfortable asking race, ethnicity and language spoken at home, and the SODH questions, it’s easier to click patient declined to answer.*” Staff
	Electronic medical/health record	“*Patients speak a myriad of languages, which is fantastic until our EMR slightly changes the wording, which changes the mapping, and language gets dropped completely. So now, instead of having the language recorded for new patients, it says patient declined to answer*.” Staff
	Data infrastructure	“*I mostly focus on prevention, so colorectal, breast, and cervical prevention screening. Those have definitely been our hardest measures since I’ve started. For breast and colorectal, they involve an external partner, which requires data sharing, which makes everything harder. We’ve had tons of challenges to overcome around that data and results piece, really have to have sophisticated processes and software. All that stuff has to just be on par to be staying on top of it. And we’ve just had antiquated systems around all of that*.” Leader
	Patient-provider communication	“*It’s hard to be accommodating culturally and linguistically to all these different communities. We have interpreters that speak seven different languages. And interpreters for 30 more. But when you’re working through interpreters and when you’re speaking to a culture that’s not your own, it just takes longer. And I think it takes more than twice as long to be understood and to understand the choices that someone might be making about their health.*” Leader
Resources and policy and practice innovations to advance health equity at FQHCs	Data infrastructure	“*I think from a general primary-care standpoint, there’s more that we could do with the right resources and ability to pull that data and create that report and analyze it and have it be meaningful, so that it really guides our direction. I think that there is some of that work that happens at a higher level, maybe more at the executive team level, that may guide some program development*.” Staff
	Training	“*I think resources in terms of all-staff trainings on the bigger concepts of what health equity is, I think that’s crucial, and we don’t have a trainer here. We don’t have people who are dedicated to that*.” Staff
		“*What would be helpful to start off with is trainings in terms of the [health equity] framework, because we can have discussions, but sometimes discussions can be damaging … How does it make one of the nurses feel when they find out that the zip code that they live in has such a high infant-mortality rate, they’re about to give birth?*" Leader
	Guidance	“*If there were a toolkit or resource guide that helps managers walk through what would health equity be, how would that translate to day-to-day work, operationalize an idea to a project and how to measure things, that would be really helpful.*” Leader
	Staff retention and promotion	“*I think investment in staffing. Being able to retain people and allow folks to grow and advance. And we just have very marginal budgets, and if we had—reimbursements for folks who do the community health work out in the field, then there’d be different models of care that we could really leverage and promote and connect it to health equity.*” Leader
	Employing diverse staff	“*I think our staff has to reflect the community. And when our staff does that, they’re able to be the interpreters for us. Diversity at all levels is helpful, particularly when we’re thinking about outreach, our public relations team and our community health workers. In addition, the general healthcare providers are the people that are really networked best into the community in conveying our outreach message*.” Leader
	Elicit input from staff	“*The direction we want to go in, not necessarily use a top-down approach, but basically pull it from the staff because they’re the ones working with patients. They’re the ones living in the community. They see what’s happening.*” Leader
	Learning community	“*Hearing narratives of how other health centers have been able to achieve equity for different priority areas, whether cancer prevention or chronic disease. How were they able to close the and make it more equitable for patients in terms of accessing services?*” Leader

### Implementation Learning Community

Thirty-nine FQHC leaders and staff (including 13 leaders, 19 quality improvement or population health staff, 4 clinicians, and 3 attendees who did not report their roles) from 13 FQHCs attended the virtual ILC session. Participants confirmed that the qualitative data resonated with their experiences operationalizing and addressing health equity at FQHCs. Facilitated discussions during the ILC session identified key considerations for equity-focused implementation research and practice at FQHCs, including: (i) striving to address root cause social determinants of inequities using limited resources available in FQHC contexts; and (ii) developing meaningful collaboration with communities for data collection, data interpretation, and data use, and data ownership.

With respect to opportunities to advance health equity at FQHCs, ILC participants noted that while they consider training to be one part of the solution to advance knowledge, understanding, or the value of working in the health equity space, the essential or effective elements of training are unknown. Participants also emphasized the need to engage communities in owning their own data and sharing de-identified data with community leaders, including politicians to shape policy to advance health equity. There was also consensus that participating as implementation research partners can be a constructive way to advocate on behalf of patients, families, and communities.

#### Phase 2—qualitative themes

**Figure 1:**
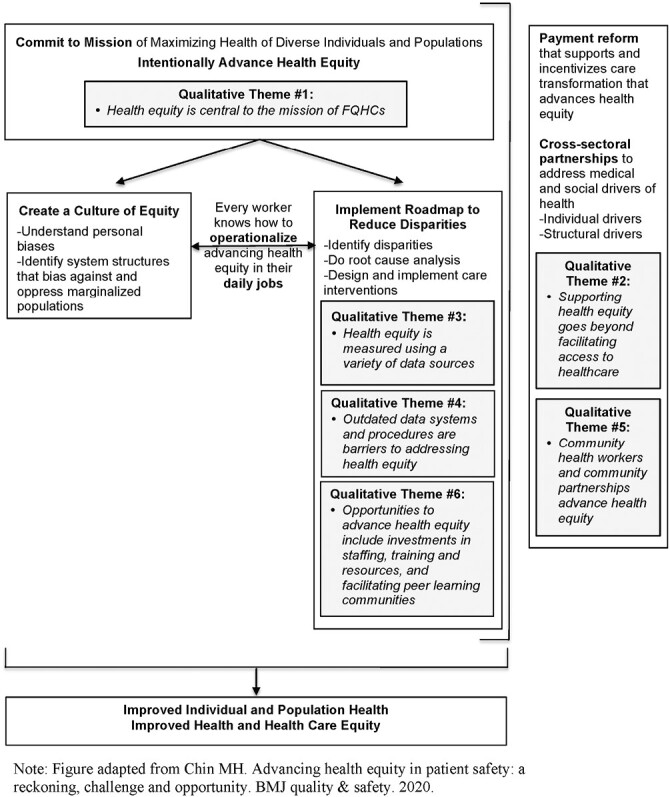
Qualitative themes mapped onto the framework for advancing health equity

Six major themes were identified from the synthesis of the phase one data, including: (i) health equity is central to the mission of FQHCs; (ii) supporting health equity goes beyond facilitating access to healthcare; (iii) health equity is measured using a variety of data sources; (iv) outdated data systems and procedures are barriers to addressing health equity; (v) community health workers and community partnerships advance health equity; and (vi) opportunities to advance health equity include investments in staffing, training and resources, and facilitating peer learning communities to share insights and lessons learned. These themes were mapped onto components of the Framework for Advancing Health Equity as illustrated in Fig. 1.

## Discussion

Our team examined how FQHC partners in our implementation science partner network defined, measured, and addressed health equity. We shared study findings with a broader group of FQHCs to identify action areas for research and practice. Facilitated discussions with FQHCs in an ILC identified key considerations for equity-focused implementation research and practice including: (i) addressing root cause social determinants of inequities using limited resources available at FQHCs; and (ii) developing meaningful collaboration with communities for data collection, data interpretation, data use, and data ownership. We identified themes in the data that we mapped onto the Framework for Advancing Health Equity including opportunities to advance health equity through investments in staffing, training, and resources. The results of this study provide clear, context-specific direction for actions to improve health equity in FQHCs and inform future approaches to health equity-focused implementation research.

Specifically, the results of this study point to three areas where implementation strategies may have an impact on advancing health equity during EBI implementation in FQHCs and other healthcare settings: (i) use of external facilitation, consultation, and/or training to help implementation partners define what it means to advance health equity in ways that align with available resources and scope of work within the organization; (ii) use of technical assistance and/or data experts to help organizations select relevant process and outcome measures of health equity; and (iii) engaging a stakeholder workgroup, including patients and families to provide input on and guidance for collaboration with communities for data collection, data interpretation, data use, and data ownership to advance health equity. We discuss each of these three areas below.

First, our FQHC partners emphasized the challenge of addressing root cause social determinants of inequities with limited resources available at FQHCs. The role of FQHCs extends beyond clinical and behavioral healthcare to include other community needs ^[29]^. Since the start of the COVID-19 pandemic, FQHCs have experienced an increased demand for non-medical social services such as housing, food, nutrition, and transportation that compliment primary care ^[30]^. Eighty-five percent of health centers responding to a national survey cited staffing shortages as a challenge in providing social and supportive services to meet patient needs ^[30]^. Future research should evaluate the use of theory-informed implementation strategies (e.g. facilitation, consultation, training) adapted to promote the equitable implementation of EBIs ^[31]^ that takes into account resource limitations (including workforce shortages) and leverages existing strengths of FQHCs. In addition, implementation strategies could be explicitly designed to facilitate, strengthen and/or sustain FQHC collaborations with systems and sectors outside of healthcare (e.g. housing, education, labor) delivering non-health interventions that address social needs to advance health equity ^[11]^.

Second, FQHC leadership and staff identified the need for guidance on the conceptualization and measurement of health equity-related projects and initiatives. External facilitation, technical assistance and/or data experts are implementation strategies ^[32]^ that could be used to help implementing organizations select relevant process and outcome measures of health equity. Our ISCCCE research team recently led the development and feasibility testing of a Stakeholder and Equity Data-Driven Implementation (SEDDI) process that supports FQHCs with external facilitation and technical assistance to use data to identify patients experiencing gaps in use of cancer control EBIs and rapidly design and test promising solutions to address gaps in service access and outcomes ^[33]^. There is ample opportunity for innovation in the use and/or adaptation of implementation science evaluation frameworks (e.g. ^[17, 34]^) to inform researcher-practice partner co-design of measures of inequities and evaluation plans to assess outcomes related to health equity.

Finally, our FQHC partners emphasized the need to engage communities in owning their own data and sharing de-identified data with community leaders (e.g. politicians) to shape public policies that will advance health equity. This position is consistent with a recent call for healthcare to play a more active role in building community power where people facing similar inequities act together to hold policy makers and institutions accountable for equitable outcomes ^[35]^. The Robert Wood Johnson Foundation established a National Commission to Transform Public Health Data Systems that recommended changing how we tell stories about the health of people and communities so equity informs meaningful narrative change; prioritizing governance of our data infrastructure to put equity at the center; and ensuring that public health measurement captures and addresses structural racism and other inequities ^[36]^.

### Limitations

This qualitative study examined how health equity was defined, measured, and addressed at FQHCs in a sample of 31 leaders and staff at 10 FQHCs in a single state. Future research should include a broader and larger sample of FQHCs in other states to increase generalizability of findings. In addition, a larger sample of FQHCs would allow investigators to stratify the data to compare and contrast findings from both FQHC leadership and staff. We did not collect demographic information from participants, which did not allow us to analyze responses within and across racial and ethnic groups that may have illuminated differences in perspectives on defining and addressing health equity at FQHCs.

We used a rapid approach to qualitative data analysis to facilitate rapid return of results to FQHC stakeholders engaged in initiatives to advancing health equity at their respective FQHCs. Rapid approaches to qualitative data analysis are designed to be less resource intensive with shorter timelines than traditional qualitative approaches ^[37]^. However, one trade-off of rapid approaches may be limited ability to compare findings across projects unless findings are mapped to a framework ^[38]^. In this study, we mapped findings to the Framework for Advancing Health Equity, which increases the potential to compare findings to other studies that use this framework.

## Conclusions

FQHC partners shared their own definitions of health equity, articulated how it was addressed in their practice settings, and identified constraints and opportunities for health equity-focused implementation research and practice. FQHCs identified key considerations for this work that included addressing root cause social determinants of inequities using limited resources available at FQHCs and meaningful collaboration with communities for data collection, data interpretation, data use, and data ownership. Opportunities to advance health equity included investments in staffing, training and resources, and facilitating peer learning communities to share insights and lessons learned about advancing health equity in FQHCs. Mapping qualitative themes from health equity-centered interviews with FQHC partners onto a framework for advancing health equity in healthcare organizations provided clear, context-specific direction for actions aimed at improving health and health care equity at FQHCs and other healthcare settings.

## Welfare of Animals

This article does not contain any studies with animals performed by any of the authors.
